# HoloInjection: augmented reality support for CT-guided spinal needle injections

**DOI:** 10.1049/htl.2019.0062

**Published:** 2019-11-26

**Authors:** Florian Heinrich, Luisa Schwenderling, Mathias Becker, Martin Skalej, Christian Hansen

**Affiliations:** 1 Faculty of Computer Science, University of Magdeburg, Universitätsplatz 2, 39106 Magdeburg, Germany; 2 Research Campus STIMULATE, Sandtorstrasse 23, 39106 Magdeburg, Germany; 3 Department of Neuroradiology, University Hospital Magdeburg, Leipziger Strasse 44, 39120 Magdeburg, Germany

**Keywords:** radiation therapy, phantoms, computerised tomography, needles, augmented reality, medical image processing, image registration, CT-guided spinal needle injections, minimally-invasive interventions, needle insertions, radiological imaging, spinal targets, out-of-plane needle orientation errors, high registration accuracy, out-of-plane orientation errors, in-plane orientation errors, augmented reality support, holoinjection, mixed reality glass Microsoft HoloLens

## Abstract

The correct placement of needles is decisive for the success of many minimally-invasive interventions and therapies. These needle insertions are usually only guided by radiological imaging and can benefit from additional navigation support. Augmented reality (AR) is a promising tool to conveniently provide needed information and may thus overcome the limitations of existing approaches. To this end, a prototypical AR application was developed to guide the insertion of needles to spinal targets using the mixed reality glasses Microsoft HoloLens. The system's registration accuracy was attempted to measure and three guidance visualisation concepts were evaluated concerning achievable in-plane and out-of-plane needle orientation errors in a comparison study. Results suggested high registration accuracy and showed that the AR prototype is suitable for reducing out-of-plane orientation errors. Limitations, like comparatively high in-plane orientation errors, effects of the viewing position and missing image slices indicate potential for improvement that needs to be addressed before transferring the application to clinical trials.

## 1 Introduction

The success of minimally invasive treatments like tumour ablations, biopsies or periradicular therapy is dependent on the placement accuracy of needle-shaped instruments. During such procedures, missing visual and haptic feedback is compensated by radiological imaging [1, 2]. These images are usually presented on a monitor in the proximity of the radiologist. Moreover, additional surgical navigation systems guiding the needle insertion process can be used [3, 4]. Such systems were shown to reduce the risk of complications by decreasing the number of required imaging scans and improving insertion accuracy [5, 6].

Like the radiological images, such navigation information is often presented on a monitor. Increased mental load and time pressure, as well as interrupted attention to the patient, are issues that may arise when frequently consulting spatially separated displays [7]. Augmented reality (AR) may solve this problem by providing all needed information directly at the intervention site [8]. Existing AR instrument navigation approaches often require additional cumbersome hardware devices, which are time-consuming to set up and thus may interfere with the general procedure workflow [9–11]. Especially small and short routine procedures like analgesic injections in periradicular therapy often do not benefit from navigation systems because of the consequent additional workload.

To overcome these issues of previous advances, this work presents a novel convenient to use and fast to set up the AR navigation system. The system was designed with a focus on the above mentioned periradicular therapy but can also be applied to various spinal interventions with a similar workflow. During these procedures, patients are immobilised and positioned in a prone position in a CT scanner. Injections are planned and performed in the transversal plane. Generally, the interventions are performed as follows:

- Preparation and patient positioning.
- Acquisition of image data.
- Access path planning.
- Needle insertion.
- Validation of needle position.
- Correction and revalidation if necessary (repeat until satisfactory).
- Analgesic injection.
- Needle removal and patient care.

In this work, the wireless mixed reality glasses Microsoft HoloLens (called HoloLens hereinafter) are used to support the needle insertion without interfering with the other steps. Hence, compared to existing approaches, no external tracking hardware or other devices are needed. For the first prototypical development stage, three guidance visualisations were implemented and evaluated in a phantom study. In particular, the achievable needle orientation accuracy and subjective measures were examined. Thus, the user study simultaneously evaluated the accuracy of the overall navigation system and compared the proposed AR guidance visualisation concepts. Moreover, a separate set of experiments was conducted, to analyse the accuracy of the implemented registration approach.

## 2 Related work

Previous AR instrument navigation advances examined different displaying modalities and visualisation methods. Video see-through AR experiences, created with monitors [12] and head-mounted displays [9], were developed to superimpose camera views on the injection site with needle guidance aids or radiological images. Projective AR approaches were used to project-specific navigation instructions on how to position and insert instruments [8, 13]. Optical see-through AR solutions enabled the superposition of guidance information through semi-transparent displays [10] or AR glasses like the HoloLens [14].

Heinrich *et al.* [11] analysed prevalent navigation visualisation methods and conclude that they can be vaguely clustered into see-through vision, access path and explicit navigation aids concepts. The latter was mainly adapted for projective AR and required regularly updated instrument tracking information to calculate current navigation instructions [8, 11, 13]. Besides tracking hardware, such systems also require stably mounted and sophisticatedly calibrated projectors, which may not be applicable for clinical routine.

See-through vision concepts enable the view through the patient's skin by visualising correctly registered anatomical structures or radiological images together with information on current instrument positions. Such concepts were realised for projective AR [13], video see-through AR [9] and optical see-through AR [10]. However, these systems consist of time-consuming to set up hardware devices. On the HoloLens, see-through vision concepts provide radiological images [15] and visualise correctly positioned target structures [16]. These systems provide only limited information on the actual planned needle insertion.

Access path visualisations focus less on giving direct navigation instructions or displaying the instrument around anatomical structures, but rather emphasise the position and orientation of the planned injection path. Thus, such systems usually do not require instrument tracking information. Access path concepts were analysed and compared with Chan and Heng [17] and were also adapted for HoloLens applications [18, 19]. Gibby *et al.* [20] evaluated the commercially available navigation software *OpenSight* (Novarad, USA) for needle placement tasks. The application visualised a 3D rendering of the target anatomy together with line-shaped access path visualisations, but required an inconvenient registration process, that is dependent on an extensive image data set needed for surface matching.

## 3 Methods

The clinical workflow for the targeted spinal interventions provides for a planning scan to be performed after patient positioning. Then a needle insertion path is planned with this data. In this work, AR is used to support the detection of that path in reality. Optical see-through head-mounted display devices were chosen for this task because they are portable, easy to set up and allow for an in situ visualisation of virtual content while providing an uninterrupted view on the patient. The HoloLens was selected from the commercially available choices, as it seemed the best-suited device [21]. The mixed reality glasses can be used wirelessly and thus do not interfere with the user's freedom of movement. A prototypical application was developed using the game engine *Unity* (Unity Technologies, USA).

### 3.1 Registration

To detect the planned needle insertion path, first, the HoloLens needed to be registered to the CT scanner and the planned access path. In compliance with our clinical partners, the transversal plane corresponding with the CT slice used for planning should be manually detected and placed in the virtual HoloLens coordinate system. However, the plane has to be rotated correctly, i.e. a virtual plane parallel to the CT gantry has to be found. This plane is called CT plane hereinafter.

**Fig. 1:**
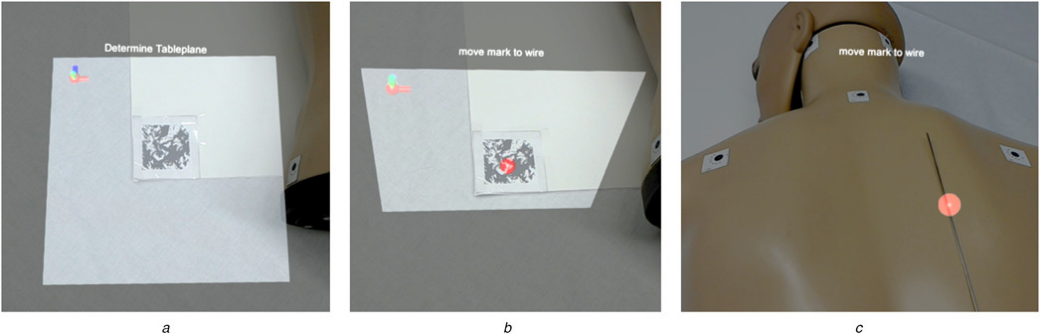
Image marker-based registration approach between the HoloLens and a desired world coordinate space, e.g. the CT scanner *a* Image marker tracking. A marker corresponding with the patient table is tracked using the *Vuforia AR SDK* *b* CT plane calculation. The CT plane, i.e. the plane corresponding with the CT coordinate system, is determined as the plane perpendicular to the tracked image marker *c* Position definition. A mark is manually moved to the respective scanning position

To this end, an image marker tracking method using the *Vuforia AR SDK* (PTC Inc., USA) was implemented. Frantz *et al.* [22] evaluated the toolkit with promising results for clinical use. To obtain the CT plane, an image marker either has to be attached to the CT gantry (thus already corresponding with that plane) or positioned on a planar reference object that can be placed on the patient table and aligned with the gantry (see Fig. 1*a*). In the second case, the CT plane is assumed to be perpendicular to the marker (see Fig. 1*b*). The markers only need to be detected once, because the HoloLens can maintain a stable world coordinate system and thus keep the relative marker position constant [23].

After finding the rotational correspondences between HoloLens and CT scanner, the CT plane needs to be translated to the respective scanning position at which the injection site is located. This is realised by a sphere that can be manually positioned using a wireless control pad. The sphere needs to be placed on the planned needle injection site for the subsequently described visualisation concepts to be displayed correctly (see Fig. 1*c*).

**Fig. 2:**
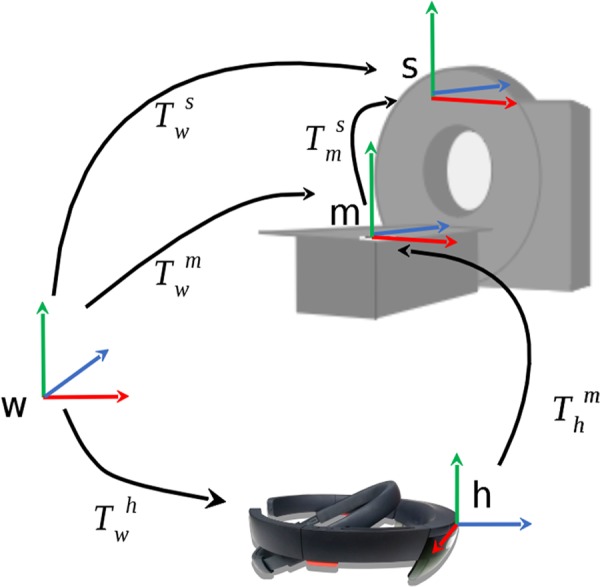
Mathematical registration model. m – image marker on patient table, s – the CT scanner, w – the world coordinate system defined by the HoloLens, h – the current HoloLens position

The mathematical model behind the registration process is visualised and described in Fig. 2 and (1) to (2). The sought-after transformation matrix between the CT-scanner and the HoloLens-defined world coordinate system ${\bi T}_{\rm w}^{\rm s} $ can be calculated by multiplying the transformation matrices ${\bi T}_{\rm m}^{\rm s} $ and ${\bi T}_{\rm w}^{\rm m} $. Thereby, ${\bi T}_{\rm m}^{\rm s} $ is manually determined during the positioning step of the registration process (see Fig. 1*c*) and ${\bi T}_{\rm w}^{\rm m} $ is given through the image marker tracking. The transformation ${\bi T}_{\rm w}^{\rm h} $ is determined through the HoloLens’ intrinsic SLAM-based spatial tracking algorithm

(1) $${\bi T}_{\rm w}^{\rm s} = {\bi T}_{\rm m}^{\rm s} \times {\bi T}_{\rm w}^{\rm m} \eqno\lpar 1\rpar $$

(2) $${\bi T}_{\rm w}^{\rm m} = {\bi T}_{\rm h}^{\rm m} \times {\bi T}_{\rm w}^{\rm h} \eqno\lpar 2\rpar $$

### 3.2 Visualisation concepts

To reduce system setup time expenditure, no external tracking hardware should be needed for the developed prototype. This limits needle tracking options to the HoloLens's front-facing RGB camera, which is already used for image marker tracking in the registration step. Such image markers could also be attached to needles [24]. However, those markers need to be of sufficient size to be reliably detectable, which may not be possible considering the small size of commonly used needles. Therefore, we decided to focus on navigation concepts, which do not require frequently updated needle position information and implemented three access path visualisations highlighting the planned injection trajectory. A visualisation of the planned injection site and insertion depth was not in the focus of this work. This information should still be obtained conventionally due to safety reasons.

**Fig. 3:**
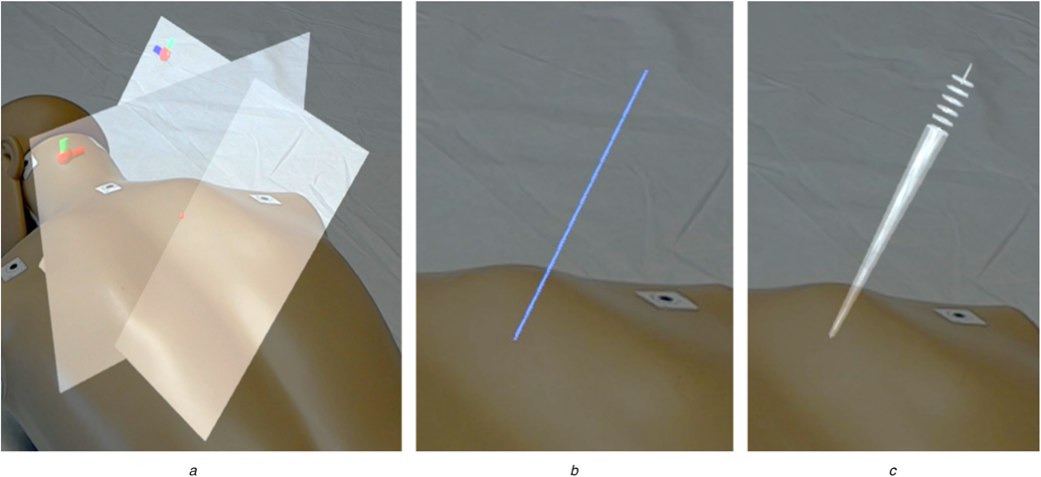
Investigated visualisation concepts as seen through the HoloLens *a* Planes concept. Perpendicular image slice plane and tilted angle plane visualise the insertion angle at their intersection site *b* Line concept. A line intersecting the skin surface at the injection site visualises the insertion angle *c* ConeRing concept. The insertion angle is visualised by a set of rings and a diffuse crepuscular strip

Fig. 3 shows the developed concepts as seen through the AR glasses. The first concept, called *Planes* concept, is based on the registration step's plane detection. Besides the CT plane, a tilted plane perpendicular to the CT gantry is visualised so that both planes intersect at the planned injection path. The *Line* concept reduces the visualised information to that path only and is comparable to the guidance visualisation used by *OpenSight* [20]. As a third concept, a method developed by Chan and Heng [17] was adapted to AR. This *ConeRing* concept was evaluated best in terms of conveying pathway information and describes the injection path as a set of rings and a crepuscular ray.

## 4 Evaluation

**Fig. 4:**
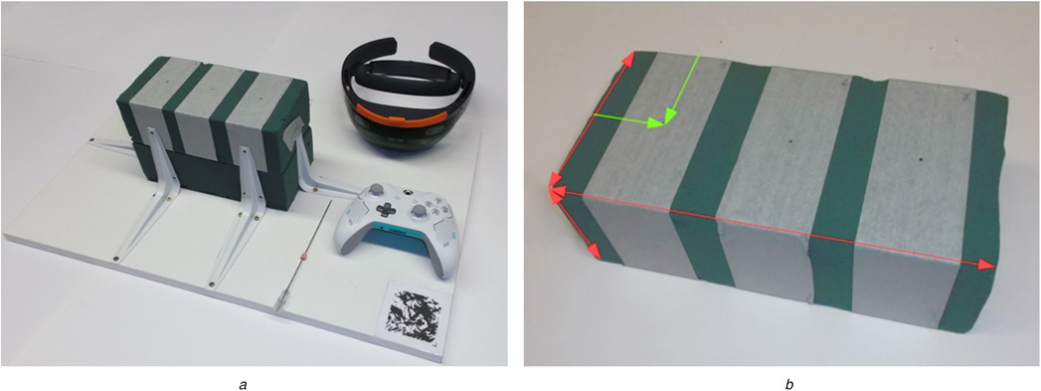
Apparatus used for comparison study *a* Registration board, floral foam bricks, needle applicator, HoloLens and control pad *b* Measured distances at a floral foam brick

After developing the described prototype, four experiments were conducted to estimate the registrations accuracy and user study to compare the developed visualisation concepts. Two of these experiments and the user study used the apparatus described in Fig. 4*a*. Two floral foam bricks were placed on a registration board with an attached image marker. A control pad was used to select injection sites on top of the floral foam bricks. Each participant calibrated the HoloLens's display to their viewing characteristics using the inbuilt calibration software before beginning an experiment.

### 4.1 Registration accuracy estimation

Four experiments were conducted to evaluate the proposed registration method. The experiments aimed to analyse the accuracy of perceived guidance visualisations and to measure angular image marker tracking accuracy.

#### 4.1.1 Angle measurement of displayed lines I

To estimate the registration accuracy, eight participants measured the perceived angle of displayed lines using a goniometer. First, they performed a registration step, as described in Section 3. They were asked to take place in front of the registration board, i.e. frontal to the CT plane. During each trial, a line tilted according to an angle from a randomised set of ten angles was visualised. The set consisted of five 5° steps from 10° upwards each clockwise and anticlockwise from the perpendicular of the board. Participants then used the goniometer to measure the angle between the displayed line and the registration board. Afterwards, the next angle from the randomised set was shown until every angle had been selected. The differences between each displayed angle and the participants’ measurements were calculated and averaged between all recorded data. This resulted in a mean deviation of $0.76^\circ \pm 0.11^\circ $.

#### 4.1.2 Angle measurement of displayed lines II

The results of the first angle measurement experiment indicate high accuracy, but only considered a frontal viewing position. To determine the effects of different viewing angles, ten participants were asked to measure the angle of lines in a similar manner. This time, each participant measured the tilting angle of five lines each for a frontal viewing position, a 45° viewing position and a lateral viewing position. Lines were tilted by a randomised angle between 30° and 80°. Angular deviations between the angles of displayed lines and measured angles were averaged for each condition. Mean deviations of $1.90^\circ \pm 1.82^\circ $ for the frontal viewing position, $4.28^\circ \pm 4.09^\circ $ for the 45° viewing position and $7.94^\circ \pm 7.75^\circ $ for the lateral viewing position indicate a clear effect of the viewing position on perceivable angle accuracy.

#### 4.1.3 Analysis of tracked normal vector accuracy I

The correct tracking of used image markers is detrimental for the accuracy of this work's proposed registration method. As a first attempt to analyse the angular tracking accuracy, the detected normal vector of a horizontally positioned planar marker was compared to the upright vector determined by the HoloLens’ gyroscope in 100 consecutive angle measurements. Between each measurement, the marker was tracked anew and after every ten repetitions, the HoloLens application was restarted to recalibrate the system's spatial mapping. Averaging the data resulted in a mean deviation of $0.72^\circ \pm 0.41^\circ $.

#### 4.1.4 Analysis of tracked normal vector accuracy II

In a second experiment to analyse the normal vector tracking accuracy, three image markers were positioned orthogonal to each other. Thus, the angles between detected normal vectors were assumed to be 90°. Deviations between that assumption and measured angles between tracked markers were recorded in 100 consecutive measurements. Again, the application was restarted after every ten repetitions in order to avoid hardware-specific bias. Calculated mean deviations varied between $0.27^\circ \pm 0.21^\circ $ between the *X* and *Y* markers, $0.31^\circ \pm 0.22^\circ $ between the *X* and *Z* markers and $0.83^\circ \pm 0.36^\circ $ between the *Y* and *Z* markers. Higher angular deviations for the latter normal vector pair may be due to image markers not have been positioned perfectly orthogonal to each other or may indicate tracking accuracy inconsistencies resulting from different viewing angle and lighting conditions.

### 4.2 Comparison study

A comparison study was conducted to determine the best suited developed visualisation concept and to overall assess the accuracy of actual needle insertions.

#### 4.2.1 Procedure

Twenty-one (21) medical students were recruited to participate in this comparison study. The sample was intended in this field because a general medical background may be helpful to assess the system generally, but no clinical experience was required for the task at hand. During the experiment, participants frequently had to insert a needle into the floral foam bricks while following the displayed guidance visualisation. The CT plane was positioned perpendicular to the bricks’ longitudinal axis. Participants were asked to stand lateral to the bricks, thus mimicking clinical workflow. Needles were inserted 10 cm deep, which was marked by a depth stop and resulted in the needles completely piercing through the first floral foam brick.

Before the actual experiment, a training phase was conducted where participants learned about the three visualisation concepts and could practise the insertion task once for each concept. After every three insertions, the top brick was exchanged and the participants were asked to track the image marker on the registration board anew. Each insertion was performed on a separate section of the floral foam bricks marked with masking tape. When a new trial began, the virtual injection site had to be moved to the centre on top of the next section using the control pad. The angles at which needles had to be inserted were randomly selected from the same set of ten angles as used in the first experiment. Then the next visualisation concept was displayed. Each concept was shown three times, once per floral foam brick. The order of concepts was randomised within each brick. After a total of nine needle insertions, the experiment concluded with an inquiry of final remarks.

For each trial, the *task completion time* was measured, which began when participants began the insertion process and ended when the needle was pulled out of the floral foam bricks. After a trial, participants were asked how easy or difficult it was to find the correct insertion angle (*subjective task difficulty*) and how confident or unconfident they were to have inserted the needle correctly (*accuracy confidence*). Both questions were answered on 6-point Likert scales. After the experiment, all floral foam bricks were measured according to Fig. 4*b*. The acquired information on the dimensions of the bricks and the relative positions of the entry and exit points of the inserted needles were then used to calculate a total insertion angle, as well as the two injection angles in the separate measurement distances. The difference between these calculated angles and the angles of visualised injection paths resulted in *total angle errors*, *in-plan orientation errors* (i.e. the angular deviation between planned and performed needle injection projected to the CT plane) and *out-of-plane orientation errors* (i.e. the angular error with which injected needles were tilted out of the CT plane).

#### 4.2.2 Results

**Fig. 5:**
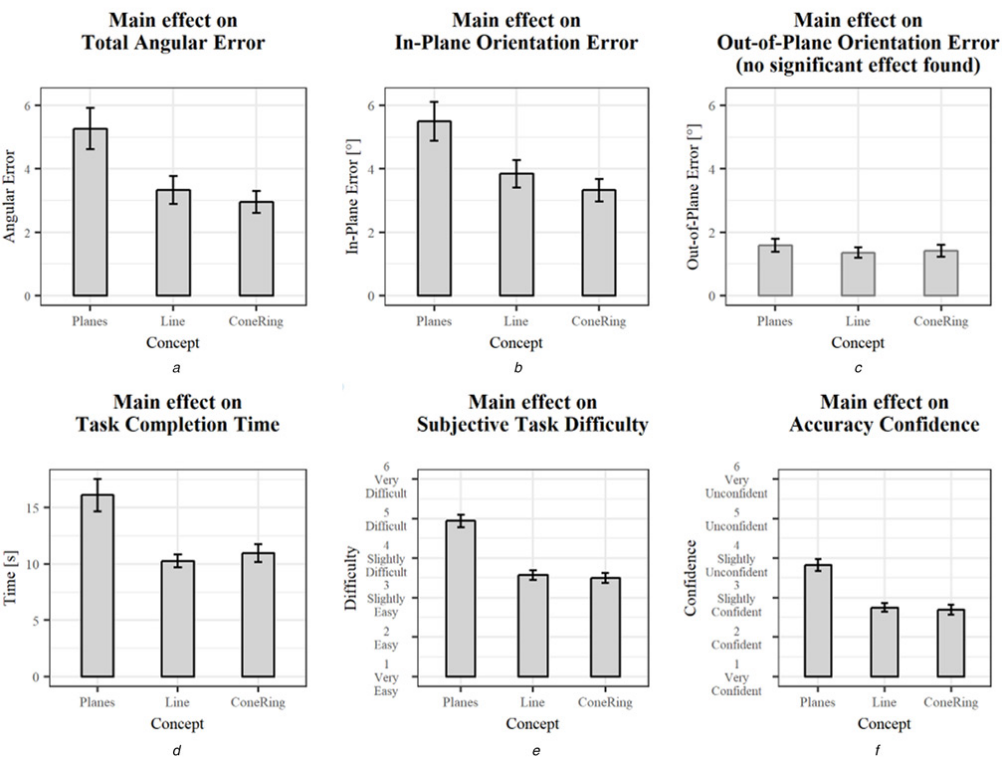
Main effects of the concept factor. Error bars represent standard error *a* Total angular error *b* In-plane orientation error *c* Out-of-plane orientation error *d* Task completion time *e* Subjective task difficulty *f* Accuracy confidence

To investigate the effects of different visualisation concepts on the regarded variables, one-way ANOVAs were conducted. These effects are summarised in Table 1 and are illustrated in Fig. 5. Data from two participants needed to be excluded from the analysis due to misunderstood instructions. During these trials, the virtual injection site was not positioned correctly, which led to falsely perceived navigation aids. This also applied to the first four trials of a third participant, but was identified and corrected during the experiment. Thus, only the data from the first trials were excluded. Additionally, inexplicable outliers were removed using the three-sigma rule, i.e. all data below or above three standard deviations from the mean value of each variable and visualisation concept combination were excluded from the statistical analysis.

**Table 1:** Summary of the ANOVA results ($\alpha \lt 0.05$)

Variable	*df*	*F*	*p*	Sig	$\eta ^2$	Effect	Figure
total angular error	2	6.35	0.002	x	0.074	medium effect	Fig. 5*a*
in-plane orientation error	2	5.57	0.005	x	0.066	medium effect	Fig. 5*b*
out-of-plane orientation error	2	0.42	0.655	—	0.005	no effect	Fig. 5*c*
task completion time	2	10.44	<0.001	x	0.117	medium effect	Fig. 5*d*
subjective task difficulty	2	35.20	<0.001	x	0.316	large effect	Fig. 5*e*
accuracy confidence	2	24.27	<0.001	x	0.232	large effect	Fig. 5*f*

Statistically significant differences between concepts were found across variables except for the out-of-plane orientation error. The ConeRing concept and the Line concept generally yielded similar results but performed better than the Planes concept. The least total angular error and in-plane orientation error were achieved using the ConeRing visualisation (resp., $2.95\, {\rm mm} \pm 2.56\, {\rm mm}$ and $3.33\, {\rm mm} \pm 2.62\, {\rm mm}$). Similar out-of-plane orientation error results range between $1.35\, {\rm mm} \pm 1.21\, {\rm mm}$ and $1.58\, {\rm mm} \pm 1.49\, {\rm mm}$ (resp., Line and Planes). Moreover, needle insertions guided by the Line and ConeRing concepts were completed faster and perceived as less difficult compared to the Planes concept. Additionally, participants were more confident in their achieved accuracy when using these concepts.

## 5 Discussion

The comparison study revealed significant differences between the evaluated visualisation concepts. The Planes concept performed worst across variables. Participants commented that they had problems perceiving the access path correctly when using this concept, which may have been due to the lateral viewing position during the experiment. From this perspective, participants had problems seeing the thin CT plane and could thus barely detect the intersection path between both planes. To improve this concept, the intersection line should be highlighted. A combination with another concept could show beneficial effects. Moreover, the CT plane could be used to display the actual CT image slice and thus create a similar experience to the work of Fritz *et al.* [10], where instrument insertions are guided by a correctly registered radiological image visible through a semi-transparent see-through display.

The overall insertion performance may have also been affected by the problem of the fixed lateral viewing position. For all concepts, a high out-of-plane orientation accuracy was achieved. However, the in-plane orientation accuracy showed comparably higher errors. The lateral viewing position may have facilitated the detection of out-of-plane angles (i.e. angles tilted left or right from the viewer) while in-plane angles were hard to perceive (i.e. angles tilted towards or away from the viewer). This is further supported by the registration accuracy results, which showed that in-plane tilted lines could be best perceived from a frontal viewing position. Since slightly diagonal viewing positions are also plausible in clinical routine, the effects of insertion accuracy results from different viewing positions should be investigated in future research.

The registration evaluation showed promising results. However, comparably high standard deviations indicate high variance in registration quality. The normal vector tracking accuracy analyses yielded feasible results below 1° angular deviation. However, even small tracking and registration errors affect the overall insertion accuracy. Future research should, therefore, focus on reducing these errors and increasing the methods robustness.

Besides being caused by registration problems, orientation inaccuracies can also mean that investigated concepts did not convey insertion information as precisely as desired. Both the Line and the ConeRing concepts allowed for ambiguities since virtual contents were displayed with a larger diameter than the needle used for insertion. However, smaller renderings may be harder to perceive. More experiments should be conducted to better understand how changes in size parameters influence insertion accuracy.

Hardware specific constraints may have also influenced the experiment. Some participants mentioned that parts of the visualisations were not visible due to the HoloLens's field of view size. Moreover, the mixed reality glasses were designed for virtual content placed at 2 m distance. Placing objects nearer than that may cause depth perception problems, which may have contributed to errors during the experiment. Different solutions suggest using a rotatable laser unit mounted at the front of the CT gantry projecting a correctly oriented laser line onto the injection site [3, 6]. These systems were shown to yield better alignment accuracy results, but restrict the radiologists’ movement area and the patient positioning to the front of the CT gantry. Wiercigroch *et al.* [25] developed a navigation tool consisting of a guiding rail for needle stabilisation, a goniometer to adjust the guiding rail's angle according to the planning data and a spirit level to correctly position the tool at the injection site. They measured less in-plane but higher out-of-plane orientation errors, resulting in similar overall angular errors. However, compared to this work's HoloLens-based method, increased infection risk may be caused by the additional patient contact from the introduction of the navigation tool.

## 6 Conclusion

This work presented the development and evaluation of an AR instrument navigation prototype to support the detection of planned insertion paths for needle-based spinal interventions. Using the mixed reality glasses HoloLens, three visualisation approaches and a method to semi-automatically register them to the patient were implemented.

An evaluation of the registration accuracy revealed viable results. However, a user study evaluating needle insertion accuracies of the different concepts revealed limitations caused by issues originating from the effects of different viewing positions, missing image slice information and comparably significant in-plane needle orientation errors. These inaccuracies have to be reduced before the prototype can be transferred to clinical trials. User errors due to the guidance visualisation and registration problems need to be addressed by future research. However, limitations intrinsic to the HoloLens, e.g. small field of view and depth perception problems, can only hardly be attenuated without replacing the hardware system. Eventually, less out-of-plane orientation errors, the registration accuracy estimation results and statistically significant results regarding the comparison of visualisation concepts constitute a promising base for further development.
